# Gastro-Colic Fistula Due to Chronic Gastric

**Published:** 1916-10

**Authors:** George M. Niles

**Affiliations:** Gastroenterologist to Georgia Baptist Hospital; Consulting Gastroenterologist to the Anti-Tuberculosis Association, Etc. Atlanta, Ga.


### Journal-Record of Medicine

Successor to Atlanta Medical and Surgical Journal, Established 1855
and Southern Medical Record, Established 1870
OWNED BY THE ATLANTA MEDICAL JOURNAL COMPANY
PUBLISHED MONTHLY.

*Official Organ Fulton County Medical Society, State Examing Board, Atlanta, Birmingham and Atlantic Railroad Surgeon's Association, Chattahoochee Valley Medical and Surgical Association, Etc.*

A. W. STIRLING, M. D., C. M., D. P. H.; J. S. HURT, B. Ph., M.D.
GEO. M. NILES, M. D., W. J. LOVE, M. D., (Ala.); Associate Editors
E. W. ALLEN, Business Manager
COLLABORATORS
W. F. WESTMORELAND, M. D., General Surgery
F. W. McRAE, M. D., Abdominal Surgery
ALLEN H. BUNCE, Medical Societies
E. B. BLOCK, M. D., Diseases of the Nervous System
MICHAEL HOKE, M. D., Orthopedic Surgery
CYRUS W. STRICKLER, M. D., Legal Medicine and Medical Legislation
E. C. DAVIS, A. B., M. D., Obstetrics
E. G. JONES, A. B., M. D., Gynecology
ALLEN H. BUNCE, M. D., Pathology and Bacteriology
R. T. DORSEY, JR., B. S., M. D., Medicine
L. M. GAINES, A. B., M. D., Internal Medicine
GEO. C. MIZELL, M. D., Diseases of the Stomach and Intestines
L. B. CLARKE, M. D., Pediatrics
EDGAR PAULLIN, M. D., Opsonic Medicine
THEODORE TOEPEL, M. D., Mechano Therapy
R. R. DALY, M. D., Diseases of the Eye, Ear, Nose and Throat
E. G. BALLENGER, M. D., Diseases of the Genito-Urinary Organs

VOL. LXIII. ATLANTA, GA., OCTOBER, 1916. NO. 7

GASTRO-COLIC FISTULA DUE TO CHRONIC GASTRIC

### THE ENEMA.

### An Humble but Invaluable Agent.

By George M. Niles, M. D.,

Gastroenterologist to the Georgia Baptist Hospital; Consulting Gastroenterologist to the Anti-Tuberculosis Association, Etc., Atlanta, Ga.

The various methods of injecting fluid into the bowel came under this term, though there are many variations in method and indication.

The principal methods consist of:

1. The simple enema, where fluid is injected into the lower bowel.

2. Irrigation with a single tube.

3. Irrigation with a double-current tube or other special appliance.

4. Proctoclysis by the drop method of injection.

There are quite a number of indications for the employment of enemas, or intestinal irrigation, some of which are:

For local treatment of diseased conditions of the gut, as catarrhal colitis.

In proctitis, prostatitis, or any acute inflammation in the pelvic region.

For the relief of pain and irritability in the anal region, as in spasm of the sphincter.

To aid in the absorption of inflammatory products in the pelvis, as of post-uterine adhesions.

To replace the loss of fluid in the body, as in cholera, or after severe hemorrhages.

To dilute the poison of disease and promote diuresis, as in uremia.

To check hemorrhage by the local effect of fluid either very cold or very hot, as in hemorrhage from ulcers in the rectum.

To assist in emptying the bowel, either by direct irrigation, or by the presence of the fluid, to stimulate the gut to expulsive efforts, as in constipation, or obstipation from retained masses of the hardened feces.

To affect the heat centers, as by hot irrigations in lowered temperature from shock, or cold irrigations in high fever.

To exert an antispasmodic effect, as in colic.

To aid in the expulsion of gas, as in excessive tympanites.

To exert a mechanical effect, as in intussusception.

To employ the fluid as a vehicle for the introduction of nourishment, as in nutritive enemata.

There are few simple mechanical procedures in the realm of therapeutics that admit of the display of more tact, ingenuity and skill than in the administration of enemas. To inject into the bowel a sufficiency of fluid to meet a given indication, without pain or discomfort to the patient, so that it can be retained long enough to accomplish the desired purpose, is not such an easy matter as some would suppose.

The necessary apparatus for enemas may consist of a 1 to 4-quart fountain syringe of rubber, or an irrigating jar of glass or porcelain with an opening at the bottom. This is connected with rubber tubing which may have at its end nozzles of various sizes and shapes, or the part intended to be introduced into the bowel may consist of a tube with recurrent flow, or a soft-rubber catheter.

Either hard-rubber nozzles or soft-rubber tubes are preferable, as, in the injection of hot fluids, a metal nozzle becomes unduly heated and uncomfortable to the patient.

The amount of hydrostatic pressure to be exerted requires judgment. In irritable conditions of the intestinal mucosa, the flow should be slow and gentle, perhaps frequently interrupted, so that the sensitive bowel will not spasmodically contract, and expel the fluid too often. Under such conditions, the container need be only 1 or 2 feet above the buttocks of the patient. Ordinarily the bag or container may be held or hung from 2 to 5 feet above the patient. Higher than that, unless extreme hydrostatic pressure is desired, as in intussusception, is fraught with danger. I might state, however, that Dr. A. B. Jamison, who has had much experience in intestinal irrigation, permits in many of his patients the water to enter at "hydrant pressure," claiming that in no instance has harm been inflicted. Individually, I would hesitate to recommend such force.

**Quantity.**—The amount of fluid to be injected depends upon the results desired. To simply stimulate lagging peristalsis, a pint, or even less, is usually sufficient. Many individuals are slightly inclined toward constipation, and need only a gentle stimulus to "wake," as it were, intestinal contractions. Many of these have in the toilet a convenient fountaint syringe, which is brought into use, should the regular daily evacuation be tardy. The employment of a small enema of warm water under such circumstances causes practically no disturbance of the alimentary tract, and is greatly preferable to the constant and promiscuous self-administration of laxatives.

Enemas intended to flush the colon, or to dislodge fecal accumulations higher up, may consist of a quart and a half, or even two quarts. The last I consider a maximum. The practice of introducing into the bowel vast quantities of water—1 or 2 gallons, or even more, is reprehensible, and liable to cause dilatation, with later on paralysis of the bowels.

A number of years ago a well-meaning but ill-informed individual promulgated a theory that, by frequently flushing the colon with enormous quantities of warm water, many of the ills of life might be mitigated or cured. This fad, like many others containing some elements of truth, became quite popular for a season, to the great harm of some of its devotees. As a sad commentary, it is reported that the originator himself died from the effects of dilated and atonic bowels.

Let me insist that several enemas of 1 quart each are infinitely better than one enema of several quarts. If this study convinces its readers of this one basic fact, my efforts will be well repaid.

Many times, if the first enema is fruitless, the water returning clear, if repeated one or more times, peristalsis will be set up, the hardened contents will in the meanwhile be softened, and satisfactory fecal results will ensue. The mere fact of repeated injections need cause no more apprehension than the mere fact of repeated ablutions to the surface of a soiled and crusted skin.

**Temperature.**—Cold enemas are indicated only in the presence of hemorrhage or hyperpyrexia. Their use is limited, and generally, any benefit which might be attained by the injection of cold fluid into the bowel, is more comfortably and safely accomplished by other means.

Generally speaking, the fluid should be about the body temperature—perhaps a little warmer. For the relief of inflammation in the intestinal mucosa or adjacent structures, the water may be quite hot. Albright advocates a temperature of 120 degrees F., while Jamison advises a temperature of 135 or even 149 degrees F. I would hardly advise an irrigation with a temperature above 125 degrees F.

The irrigating tube must never be removed while the hot solution is in the rectum, as should it come in contact with the anus, it would cause decided pain. It must be remembered that the interior of the rectum will comfortably bear a degree of heat that the anus cannot endure, so the instrument should not be withdrawn until after the fluid ceases flowing through it, and then slowly.

**Lubrication.**—It is always conducive to the comfort of the patient that the nozzle, the entering tube, or the colon tube be well lubricated. Vaseline, olive oil, caston oil (warmed), or even toilet soap will answer the purpose. Laundry soap, or the cheap grades of turpentine soap are useful in the water, but are unsuitable to lubricate a tube that passes over a possibly tender or excoriated surface.

**Preparation of the Irrigating Fluid.**—A simple enema for gentle stimulation of peristalsis may consist of warm water alone.

The so-called S. S. enema consists of warm water into which sufficient soap is rubbed to form a liberal amount of soapsuds. In such an enema laundry—or turpentine—soap may be used, as this soap exerts a slightly stimulating effect.

The saline enema (normal) consists of one teaspoonful of common table salt to the pint of water.

An oxgall enema contains one teaspoonful of oxgall to the pint of water.

The Hare enema consists of magnesia sulphate, one tablespoonful, glycerin, 1 ounce, water, 2 quarts.

Various carminative enemas may be prepared by adding to the water one or more tablespoonfuls of milk or asafetida, to the quart of water, one teaspoonful of powdered alum or powdered borax to the quart, or a weak infusion of camomile.

Emollient enemas contain corn starch in sufficient quantity to thicken the fluid; or flaxseed meal or slippery elm bark, with the water strained. Gum arabic or tragacanth is also used.

Antiseptic enemas may contain permanganate of potash, one to two or five thousand, nitrate of silver, 15 grains to the quart, phenol, 30 grains to the quart (being sure that it is all returned), chlorinated lime, half teaspoonful to the quart, commercial sulphuric acid, one-half dram to the quart, or the liquor alkaline antiseptic (N. F.) 1 or 2 ounces to the quart.

For softening and healing enemas there may be employed several of the oils. These are also employed in the treatment of constipation, and, when rightly used, are successful in a large percentage of cases. For healing an irritated intestinal mucosa, there may be added to the oil a small amount of phenol, 1 dram to the pint, tincture of iodin, the same amount, or bismuth subnitrate in any quantity desired, so the oil is not made too thick by its addition. For inflammatory conditions, when pain or tenesmus is present, and the oil is not expected to remain in the bowel any great length of time, the amount injected may vary from 8 ounces to a quart, or even more.

In constipation, the method is different. The oil should be placed in a glass or hard-rubber irrigating jar, as its frequent use rots the bag of a fountain syringe. Not over 3 ounces should be injected the first time, though, as the patient finds the bowel will retain more, this amount may be increased up to 8 ounces. When injecting the oil, the bed should be protected by a rubber sheet or other covering. The patient should lie on his left side with his legs flexed and his hips slightly elevated. The rectal tube is slowly introduced, and, as the oil flows in, is gently pushed up until it enters as much as 4 or 6 inches. After the oil flows in the tube is withdrawn, and compressed by the finger during its exit to prevent the escape of random drops. The patient should remain on his left side for twenty or thirty minutes, and, if possible, the oil should remain in all night. This usually is accomplished, except in rare instances of extreme irritability of the rectum, or where the anus is patulous, allowing it to escape during sleep. This injection of oil is generally followed by a satisfactory evacuation of the bowels the following morning; but, if not, a small S. S. enema, or a glycerin suppository, will set up enough intestinal contractions.

This method is specially applicable to those forms of constipation characterized by hard and dry fecal masses, with a tendency to accumulation of scybalous collections high up in the large intestine.

**The Kind of Oil to be Used.**—Some writers advise the pure olive oil, which is both expensive and hard to obtain in many instances. Hemmeter has observed occasional irritation from fatty acids in the oil, and advises shaking the oil with hot water, as the latter takes up the fatty acid. Rosenheim adds a little bicarbonate of soda to neutralize the acid. Either of these procedures I have never found necessary or expedient. Linseed oil has been advocated by some, but when warm it is so fluid, that it tends to run out of the bowel, unless the sphincters are quite efficient.

The best and most satisfactory oil in my experience is the cotton-seed oil, especially after it has been refined for cooking purposes. The various cooking oils are cheap, easily obtained at the nearest grocery store, and answer every purpose that can be attained by pure and expensive olive oil.

When it is considered that most of the so-called olive oil now on the market is adulterated with cotton-seed oil, the reader may see that it is unnecessary to have the patient pay a large price for supposed olive oil, when the pure cotton-seed oil is fully as suitable for the desired purpose, and much cheaper.

**The Colon Tube.**—This, as far as effective results are concerned, is a fallacy and a delusion. The writer has demonstrated by the Roentgen ray, that the colon tube, instead of following the course of the colon, curls up in the ampulla of the rectum; furthermore, it has been thoroughly demonstrated that by employing only a simple tube, extending past the internal sphincters, an opague enema may be sent throughout the entire colon, filling the cecum, and, in the presence of incompetence of the ileocecal valve, progressing into the ileum. Away with the colon tube!

**Irrigation of the Large Intestine.**—This is accomplished by several methods. To irrigate with a single tube, a colon tube about 2 feet long, which is surmounted by a funnel, as in gastric lavage. This method is not very satisfactory, and is useful only to remove softened feces or small shreads of mucus, or in the absence of better appliances.

The best and most scientific methods of irrigation lie in the several forms of recurrent tubes, in which the water flows out as fast as it enters, there is no straining or tenesmus, and the temperature of the fluid can be absolutely regulated. By this method also an unlimited quantity of the irrigating fluid may be made to leave the intestines, and besides mechanically cleaning them, the flatus is relieved by the suction of the return flow.

I prefer the Kemp flexible recurrent irrigator and the Albright small hard-rubber irrigator, though Tuttle, Hemmeter and several others have devised successful instruments for this purpose.

The principle is simple, being a double, rigid tube, where the fluid enters by a central tube, while the outflow is carried off by the outer tube into which the fluid flows by lateral orifices.

In using the double-current irrigating tube, the patient may be either on the back or side, just so the hips are elevated. The height of the douche bag or irrigating jar should be from 3 to 5 feet above the patient. There are several precautions advisable, which will facilitate every step of the irrigation. Allow the fluid to flow from the tube before insertion, so as to force out the air, and then check the flow, then renew the flow as the tip of the instrument passes well through the spincters, so as to force the mucosa away from the irrigator and lateral fenestrae.

The instrument should be well lubricated (the flowing fluid will warm it), inserted with a gentle rotary movement with the tip directed slightly back toward the sacrum. Do not use force in entering, nor press the tip of the tube against the intestinal walls. Should the flow cease, rotate the tuble slightly, or withdraw it some while rotating, and then push it gently backward and forward till the flow resumes. Occasionally, where there are much hardened fecal contents present in the bowel, the larger masses need to be cleared with a soapsuds enema, after previously softening them with oil or glycerin. The irrigator should be introduced to one-half its length in prostatic cases, and full length for high irrigation.

Should the tube encounter an obstruction, a rectal examination will disclose the cause, such as a possible enlarged prostate, uterine fibroids, redundant hemorrhoids, etc. These, however, can generally be passed with the tube, after their location and size are known. When withdrawing, bring out the tube with a gentle rotary pull, lest the mucosa catch in the fenestrae.

When it is desired to thoroughly irrigate the whole colon, the irrigation may be started with the patient on the left side with his hips elevated; as the irrigation proceeds, he is gently rotated to the dorsal position, then to his right side. As the irrigation is still kept up, he may be rotated in the opposite direction, and the procedure concluded after he is returned to the left side. It is also of assistance to raise the shoulders while on the right side, as this tends to make the fluid gravitate into the caput coli.

**Temperature of the Irrigating Fluid.**—This may vary from 100 degrees F. to 105 degrees F. in intestinal catarrh to 110 degrees F. in typhoid fever or any toxic condition, as this higher temperature increases its eliminative effect. Jamison uses irrigation as hot as 125 to 135 degrees F.

**Solutions Employed.**—Thin flaxseed tea, normal saline solution with spirits of peppermint or cinnamon or fennel, plain water with milk or asafetida, soda, boric acid, tannic acid, tannin, or alum, the latter six medicaments being used in strength of 1 dram to the quart. Others are the solutions of silver nitrate, potassium permanganate and alkaline antiseptic liquid, as previously mentioned.

One more irrigating fluid I wish to specially mention as worthy of use—the plain kerosene or coal oil of commerce. As an arrigating fluid in chronic proctitis and colitis, where there are old and unhealthy ulcers, with superficial sloughs, and perhaps a chronic diarrhea, an irrigation of one quart on alternate days, until about three or four irrigations have been given, will in most cases yield gratifying results. There seems to be no danger of toxic effects, for in several instances quite a residue of the oil has been retained one to three hours before escaping, and in no instance have any evil or disquieting symptoms ensued. I commend this unhesitatingly.

**Proctoclysis.**—By this is meant the injection of normal saline or other solution into the rectum by the drop method, as first suggested by Dr. J. B. Murphy, of Chicago. This procedure is of special value in sepsis, and is of use as an adjunct to other treatment in post-operative shock, intentinal dilatation and atony, and uremia. In septic conditions, it is well, however, to first thoroughly irrigate the colon with a saline solution at 120 degrees F., then follow with proctoclysis.

One of the main difficulties in the administration of a solution by the drop method, is the maintenance of a constant temperature. There have been several methods suggested, but probably the most simple and satisfactory is the employment of a vacuum bottle, as directed by Kemp.

His method is as follows: Through a screw cap, which closes the bottle, passes a small hard-rubber connecting tube, to which is attached the outflow tube. Parallel with this is the filiform tube, which allows the entrance of a fine column of air, so as to render the flow possible. This last tube passes through the solution to within about one eighth of an inch from the bottom of the bottle. As the instrument is inverted when in use, it would correspond to the same distance from the top of the bottle. This filiform tube is of hard rubber externally where exposed to the air, as a non-conductor of heat. The part lying within the bottle is purposely made of metal, so that it is rapidly heated from the surrounding solution, and the entering air is thus also heated.

A series of experiments have demonstrated that there is a loss of only 1 or 2 degrees F. in the temperature of the solution in the bottle during the administration of proctoclysis lasting half an hour to an hour. The screw compression valve is applied close to the bottle attachment, so as to avoid as much as possible the solution cooling in the soft outflow tube. This outflow tube is joined to the catheter by a short piece of glass tubing for the purpose of observing whether the flow is constant. The catheter for rectal injection passes through a self-retaining rectal tip, and this catheter and tip can be inserted any length desired. The conducting tube is quite thick. An asbestos tube surrounds the conducting tube from its junction at the bottle to the catheter. This lessens the dissipation of heat, and is greatly preferable to the use of cumbersome hot towels. The asbestos wrapping can be occasionally slipped off the glass connecting joint, so as to observe the flow. The vaccum bottle is filled in the usual manner, and the special cap with attachment screwed on. The bottle is then inverted, and suspended by a sling. A small amount of fluid will escape from the bottle by the filiform air tube until the solution reaches the level of the tube, which is now near the top of the bottle. The bottle is now suspended about 6 inches above the rectum, or higher, if desired and the flow tested for proper speed before inserting the rectal tip or catheter. If flatus occur, lower the reservoir for a short time to below the level of the abdomen, so the gas may escape into the bottle. Should this not be adequate for the escape of the flatus, it may be necessary to remove the tube for a short period.

At the start the speed of the drop is more rapid, and though gauged to, say, fifteen drops per minute may, in the course of two minutes drop to five. A test of two to three minutes should be made, therefore, before inserting the catheter, so as to insure a constant flow at the desired rate. Other special apparatus for the maintenance of heat consists of gas or oil burners under the container, or electric heaters.

It will be observed from the various methods discussed in this study that a considerable portion of therapeutics directed toward alleviation of gastrointestinal ailments may be properly placed under the caption of "Local Treatment of the Large Intestine." Let me, therefore, urge my readers to not underestimate the importance of thoughtful consideration and care of the colon and rectum, for in many instances the etiologic key lies here, and here also, by the exercise of suitable measures, may be discovered a solution of the whole pathologic problem.

